# Exosome proteomic analyses identify inflammatory phenotype and novel biomarkers in African American prostate cancer patients

**DOI:** 10.1002/cam4.1885

**Published:** 2019-01-08

**Authors:** Gati K. Panigrahi, Prakash P. Praharaj, Hiroki Kittaka, Asit R. Mridha, Olen M. Black, Rakesh Singh, Roger Mercer, Adrie van Bokhoven, Kathleen C. Torkko, Chapla Agarwal, Rajesh Agarwal, Zakaria Y. Abd Elmageed, Hariom Yadav, Santosh K. Mishra, Gagan Deep

**Affiliations:** ^1^ Cancer Biology Department Wake Forest Baptist Medical Center Winston‐Salem North Carolina; ^2^ Department of Molecular Biomedical Sciences, College of Veterinary Medicine NC State University Raleigh North Carolina; ^3^ Translational Science Laboratory, College of Medicine Florida State University Tallahassee Florida; ^4^ Department of Pathology University of Colorado Anschutz Medical Campus Aurora Colorado; ^5^ School of Pharmacy University of Colorado Anschutz Medical Campus Aurora Colorado; ^6^ Department of Pharmaceutical Sciences Texas A&M Rangel College of Pharmacy College Station Texas; ^7^ Department of Internal Medicine‐Molecular Medicine and Department of Microbiology and Immunology Wake Forest Baptist Medical Center Winston-Salem North Carolina; ^8^ Wake Forest Baptist Comprehensive Cancer Center Winston‐Salem North Carolina; ^9^ Department of Urology, Wake Forest School of Medicine Winston‐Salem North Carolina

**Keywords:** biomarker, exosomes, health disparity, inflammation, prostate cancer

## Abstract

African American men face a stark prostate cancer (PCa)‐related health disparity, with the highest incidence and mortality rates compared to other races. Additional and innovative measures are warranted to reduce this health disparity. Here, we focused on the identification of a novel serum exosome‐based “protein signature” for potential use in the early detection and better prognosis of PCa in African American men. Nanoparticle tracking analyses showed that compared to healthy individuals, exosome concentration (number/ml) was increased by ~3.2‐fold (*P* ˂ 0.05) in the sera of African American men with PCa. Mass spectrometry‐based proteomic analysis of serum exosomes identified seven unique and fifty‐five overlapping proteins (up‐ or downregulated) in African Americans with PCa compared to healthy African Americans. Furthermore, ingenuity pathway analyses identified the inflammatory acute‐phase response signaling as the top pathway associated with proteins loaded in exosomes from African American PCa patients. Interestingly, African American PCa E006AA‐hT cells secreted exosomes strongly induced a proinflammatory M2‐phenotype in macrophages and showed calcium response on sensory neurons, suggesting a neuroinflammatory response. Additionally, proteomic analyses showed that the protein Isoform 2 of Filamin A has higher loading (2.6‐fold) in exosomes from African Americans with PCa, but a lesser loading (0.6‐fold) was observed in exosomes from Caucasian men with PCa compared to race‐matched healthy individuals. Interestingly, TCGA and Taylor's dataset as well as IHC analyses of PCa tissue showed a lower Filamin A expression in tissues of PCa patients compared with normal subjects. Overall, these results support the usefulness of serum exosomes to noninvasively detect inflammatory phenotype and to discover novel biomarkers associated with PCa in African American men.

## 1 INTRODUCTION

Prostate cancer (PCa) is the most diagnosed noncutaneous cancer and second leading cause of cancer‐related deaths in men and displays a distinct racial disparity.^1^ Risk factors for the disease are age, family history, race/ethnicity, and inherited genetic factors that include about 100 known disease susceptibility loci.^2^, ^3^ African American men have a mortality rate more than double to Caucasian men in part because of their increased risk to develop PCa.^4^ Other reasons for PCa‐related disparity are that presently we lack effective biomarkers to predict PCa aggressiveness and progression in African Americans.^5^ For example, African American men with Gleason score 6 PCa produce significantly lesser prostate‐specific antigen (PSA) and have significantly lower PSA density than Caucasian men. As a result, active surveillance criteria predictive in Caucasian men are not accurate in African American men.^6^ Clearly, additional and effective biomarkers are needed for better disease prognosis in African American men.

Inflammation is considered as one of the causative factors in prostate carcinogenesis^7^, ^8^ and is associated with aggressive disease.^9^, ^10^, ^11^ Several studies have described distinct immune‐inflammation signatures in prostate tumors from African American patients.^12^, ^13^, ^14^, ^15^ Race/ethnic differences in prostatic inflammation also exist in noncancerous tissues.^16^ Studies have suggested that inflammation is more prevalent in nontumor prostate biopsy specimens from African American men when compared with Caucasian men.^17^ Consistent with these observations, it has recently been reported that anti‐inflammatory drugs like aspirin reduce the risk of aggressive PCa and disease recurrence in African American men.^18^ Accordingly, it is important to develop noninvasive biomarkers to detect inflammation in PCa patients for better prognosis and possible intervention using anti‐inflammatory agents.

Exosomes are small vesicles (~30‐150 nm in diameter) originating from endosomes and secreted into the extracellular milieu after the fusion of multivesicular endosomes (MVEs) with the plasma membrane. There are several physiological conditions and molecular players that regulate exosome biogenesis. For example, oxidative stress and hypoxic conditions have been known to promote exosome biogenesis.^19^, ^20^ Recent literature has established a critical role for these nano‐sized vesicles in intercellular communication, tumor growth, and progression.^21^ Importantly, exosomes are also emerging as important tools for disease diagnosis and prognosis.^22^, ^23^, ^24^, ^25^ McKiernan et al developed a urine exosome‐based assay for better prognosis of the disease.^25^ Importantly, exosomes could predict the physiological state of cancer cells and tumor microenvironment.^26^, ^27^ In the present study, we focused on characterizing exosomes from the serum of PCa patients (African American and Caucasian) and conducted an untargeted analysis of exosomal proteins to identify race‐specific biomarkers. The proteomic analysis revealed a higher expression of Filamin A in African American men with PCa. Filamin family of proteins is essential for mammalian cell locomotion and migration,^28^ and several studies have indicated the correlation of Filamin A expression with cancer stages and patient prognosis.^29^ Therefore, we also characterized Filamin A expression in available PCa datasets as well as in archived PCa tissue sections from both African American and Caucasian men.

## 2 MATERIALS AND METHODS

### 2.1 Human serum samples, cell lines, and reagents

Serum samples from African American and Caucasian PCa patients and matched healthy donors were collected after patients’ consents following an IRB‐approved protocol at University of Colorado Anschutz Medical Center, Colorado. Human PCa E006AA‐hT cells were obtained from Dr Koochekpour at Roswell Park Memorial Institute. RAW 264.7 cells were provided by Dr David R. Soto‐Pantoja, Wake Forest Baptist Medical center. RPMI1640, penicillin and streptomycin, 0.25% trypsin, and fetal bovine serum (FBS) were obtained from Gibco Laboratories (Gaithersburg, MD). Primary antibodies for Filamin A and arginase were purchased from Abcam (Cambridge, MA). ImmPACT^TM^ Vector^®^ Red substrate was procured from Vector laboratories (Burlingame, CA). DAPI was obtained from Cell Signaling Technology (Danvers, MA). All other reagents were obtained at the highest purity commercially available.

### 2.2 Exosome isolation

Exosomes were isolated from serum by ultracentrifugation method. In brief, serum was centrifuged at 2000 *g* for 10 minutes to remove any cellular debris. Thereafter, supernatant was diluted two times using DPBS, filtered through 0.22‐µm filters, and then centrifuged at 10 000 *g* for 30 minutes. Then, the resulting supernatant was subjected to ultracentrifugation at 100 000 *g* for 90 minutes (L‐80 Ultracentrifuge, 70.1 Ti fixed angle rotor, Beckman Coulter) and the pelleted exosomes were resuspended in DPBS and stored at 4°C until further use.

Exosomes from the conditioned media of E006AA‐hT cells grown for 48 hours either under normoxia (~21% O_2_) or hypoxia (1% O_2_) were isolated by ultracentrifugation method, as reported by us previously^20^, ^30^ and labeled as Exo^Normoxic^ and Exo^Hypoxic^, respectively. In brief, the collected cell culture media was centrifuged at 300 *g* at 4°C for 10 minutes to remove detached cells. Then, the supernatant was collected and filtered through 0.22‐µm filters. The filtrate was concentrated using concentrators (150 K MWCO/20 mL, Thermo Scientific) by centrifuging at 2000 *g* for 15 minutes. The supernatants were then subjected to ultracentrifugation at 100 000 *g* for 90 minutes (L‐80 Ultracentrifuge, 70.1 Ti fixed angle rotor, Beckman Coulter). Finally, the pelleted exosomes were resuspended in DPBS and stored at 4°C until further use.

### 2.3 Nanoparticle tracking analysis (NTA)

Exosome concentration was analyzed using a NanoSight LM10 system (Nano sight Ltd, Navato, CA) equipped with a blue laser (405 nm). Nanoparticles were illuminated by the laser, and their movement under Brownian motion was captured for 60 seconds. The process was repeated three times. Then, all the three recorded videos were subjected to NTA using the Nanosight particle tracking software (Version NTA 3.1) to calculate exosome concentrations and size distribution.

### 2.4 Transmission electron microscopy

Initially, 400 mesh copper grids (formvar/carbon coated, glow‐discharged) were dipped in 100% ethanol for 5 minutes. Five to ten microliters of exosome sample (in PBS) was applied on a parafilm as a droplet. Then, the 400 mesh copper grids were put on the sample droplet in such a way that the dark side of the grid was facing toward the sample. After 5 minutes, the grid was transferred to 2.5% glutaraldehyde and incubated for 5 minutes. Then, the grid was washed three times with ultrapure water and incubated with 2% uranyl acetate for 5 minutes for negative staining. Finally, the grids were dried and viewed using TecnaiTM G2 Spirit BIOTWIN Transmission electron microscope equipped with AMT Image capture 2Vu camera system.

### 2.5 LC‐MS/MS on Dionex‐QEHF

Exosomes (50 μg) were lysed in 1× SDS buffer and run on a precast 8% polyacrylamide gel. The run was stopped as soon as all the proteins were in the resolving gel, and the gel slice was carefully excised. In‐gel digest was performed using ProteoExtract All‐in‐One Trypsin Digestion Kit (Calbiochem) according to manufacturer's instructions. Peptides were eluted with 300 µLs of 0.1% FA. Eluent was dried in a lyophilizer, and peptide mixture was fractionated using Pierce high pH reverse‐phase peptide fractionation kit according to manufacturer's recommendations. Three eluted fractions were dried and resuspended in 25 µLs of 0.1%FA, and 6 µLs was injected in each run.

An externally calibrated Thermo Q Exactive HF (high‐resolution electrospray tandem mass spectrometer) was used in conjunction with Dionex UltiMate3000 RSLCnano System. The Acclaim PepMap (RSLC 75 μmol/L × 15 cm nanoviper) C18 column was used for LC separation. The LC eluent was directly nanosprayed into Q Exactive HF mass spectrometer (Thermo Scientific). During the chromatographic separation, the Q Exactive HF was operated in a data‐dependent mode and under direct control of the Thermo Excalibur 3.1.66 (Thermo Scientific). MS data were acquired using a data‐dependent top 20 method for the Q Exactive HF, dynamically choosing the most abundant not‐yet‐sequenced precursor ions from the survey scans (350‐1700). To enable label‐free quantification, all measurements were done at room temperature and three technical replications were used for three biological replicates to enable statistical comparisons between the samples. Resultant raw files were searched with Proteome Discoverer 1.4 using SequestHT and Mascot 2.1 as the search engines using SwissProt Human fasta database and percolator as peptide validator. Protein and peptide identities were validated using Scaffold software (version 4.3.4, Proteome Software Inc, Portland, OR).

### 2.6 Ingenuity pathway analysis

Pathway analysis was carried out using Ingenuity pathway analysis (IPA) (Ingenuity Systems, USA) software package. Identified proteins were functionally assigned to canonical pathways and subsequently mapped to the most significant networks generated from previous publications and public protein interaction databases. A *P* value calculated with the right‐tailed Fisher's exact test was used to yield a network's score and to rank networks according to their degree of association with our dataset.

### 2.7 Cell culture and hypoxia exposure

E006AA‐hT cells were grown in RPMI1640 medium supplemented with 10% FBS and 100 U/mL penicillin G and 100 µg/mL streptomycin sulfate. All cells were cultured at 37°C in a 5% CO_2 _humidified environment as an adherent monolayer; this represented normoxic conditions (21% O_2_). Hypoxia experiments were performed in a hypoxia chamber (Baker Ruskinn INVIVO_2_ 400) at 1% O_2_ at 37°C in a 5% CO_2_ humidified environment.

### 2.8 Immunofluorescence/confocal microscopy

THP1 cells (monocytes) (10 000 cells/well) were seeded in chamber slides (Nunc™ Lab‐Tek™ II Chamber Slide™ System) in RPMI‐1640, supplemented with 10% FBS and 1% penicillin/streptomycin in the presence of PMA (100 ng/mL) for 12 hours, with the aim of differentiating monocytes to macrophages. Thereafter, cells were treated with Exo^Normoxic^ or Exo^Hypoxic^ (10 μg each) for 48 hours The effect of exosomes treatment on the THP1 macrophages polarization state was then assessed by arginase‐1 expression (a M2 phenotype marker protein) through confocal microscopy as described earlier.^31^ In brief, cells were incubated with primary antibody (anti‐arginase) followed by secondary antibody conjugated with Alexa Fluor, followed by staining with Prolong^®^ Gold Antifade Reagent with DAPI. Fluorescent images were captured using an Olympus FV1200 SPECTRAL Laser scanning Confocal Microscope at 60× objective lens (Olympus IX83 inverted platform).

### 2.9 Primary neuron culture

Dorsal root ganglia (DRG) were rapidly dissected on ice from adult C57BL/6J mice after CO_2_ euthanasia and then dissociated by incubation for 30 minutes at 37°C in Dulbecco's modified Eagle's medium (DMEM; Mediatech, Inc, VA, USA) containing 10% fetal bovine serum (FBS; VWR International, LCC., PA) and 50 units/mL penicillin/50 μg/mL streptomycin with 2.5 mg/mL collagenase (C7657; MilliporeSigma, Burlington, MA, USA). After changing the solution, DRGs were gently triturated with a fire‐polished Pasteur pipette in DMEM. Collected DRG neurons were inoculated in a drop of DMEM on 18‐mm glass coverslips (VWR) precoated with 15 μL/slip of 0.4 μg/mL laminin and 0.01% poly‐l‐lysine (MilliporeSigma) and incubated for 30 minutes at 37°C in 5% CO_2_ followed by DMEM addition. Cultured DRG neurons were used for Ca^2+^ imaging experiments 24‐48 hours after dissection.

### 2.10 Ca^2+^ imaging

For Ca^2+^ imaging experiments, cultured DRG neurons for 24‐48 hours were incubated for 30 minutes in DMEM containing 1 μmol/L of the fluorescent indicator Fura‐2 AM (Enzo Life Sciences, Inc, NY). Fura‐2 fluorescence was measured under conditions where a coverslip was set in a recording chamber that was then perfused with a standard bath solution containing 140 mmol/L NaCl, 5 mmol/L KCl, 2 mmol/L MgCl_2_, 2 mmol/L CaCl_2_, 10 mmol/L Hepes, and 10 mmol/L d‐glucose at pH 7.4 adjusted with NaOH. Fura‐2 fluorescence was excited at 340 and 380 nm, and emission was monitored at 510 nm with a digital CCD camera (Andor Co., Ltd, London, UK). Data were obtained every 5 seconds using a software (NIS‐Elements AR 4.13.04 64‐bit, Nikon Corporation, Japan) and analyzed using Microsoft Excel (Microsoft, WA, USA). The 340/380 ratio value (described as *F*) was calculated using regions of interest that included the whole cell body of single DRG neurons. The changes in the 340/380 ratio values (*F*/*F*_0_) were calculated using *F*_0_, averaged *F *value for the first 25 seconds of respective DRG neurons in experiments. In this study, changes in each response to an application with *F*/*F*_0_>0.1 were regarded as positive. Cells not responding to 100 mmol/L KCl, which was applied in the last of each experiment, were regarded as non‐neuronal cells and excluded from analysis.

### 2.11 Filamin A expression in prostate cancer datasets

The expression of Filamin A in prostate adenocarcinoma (PRAD) TCGA samples was carried out using UALCAN interactive web resource (http://ualcan.path.uab.edu/). The expression of Filamin A in Taylor's cohort was carried out using Gene Cluster Text file (.gct) generated from Taylor's cohort (GSE21034) by separating PCa patients based on their major cancer stages (normal, primary tumor, and metastasis).

### 2.12 Immunohistochemistry

Immunohistochemistry (IHC) for Filamin A was performed on formalin‐fixed paraffin‐embedded tissue sections (4 µm thick) from prostatectomy specimens. Antigen unmasking was performed by heat‐induced epitope retrieval using citrate buffer (10 mmol/L) at pH 6.0, and endogenous peroxidase activity was quenched by incubating with BLOXAL blocking solution for 10 minutes. Protein block was done by normal horse serum (2.5%) for 20 minutes. Then, the sections were incubated with HRP conjugated anti‐Filamin A antibody (rabbit monoclonal) (1:400) overnight at 4°C, followed by anti‐rabbit secondary antibody for 30 minutes at room temperature. ImmPACTTM Vector^®^ Red substrate (Vector Laboratories, Burlingame, CA) was used for stain development, and counterstain was done with hematoxylin. Microscopic examination was done to confirm the diagnosis and tumor grade. All the immunostained slides were scanned by NanoZoomer (Hamamatsu, Japan) using 40× lens, and scoring was assigned to each case on a scale of 0 to 3 with 0 being no staining, 1+, weak staining; 2+, moderate staining; and 3+, strong staining.

### 2.13 Statistics

Statistical analysis was performed using GraphPad Prism (Version 5.00, GraphPad Software Inc, La Jolla, CA) and presented as mean ± SEM. A two‐sample *t* test was performed, and a *P* value of ≤0.05 was accepted as statistically significant.

## 3 RESULTS

### 3.1 PCa patients have higher amount of exosomes in their blood compared to healthy men

**Figure 1:**
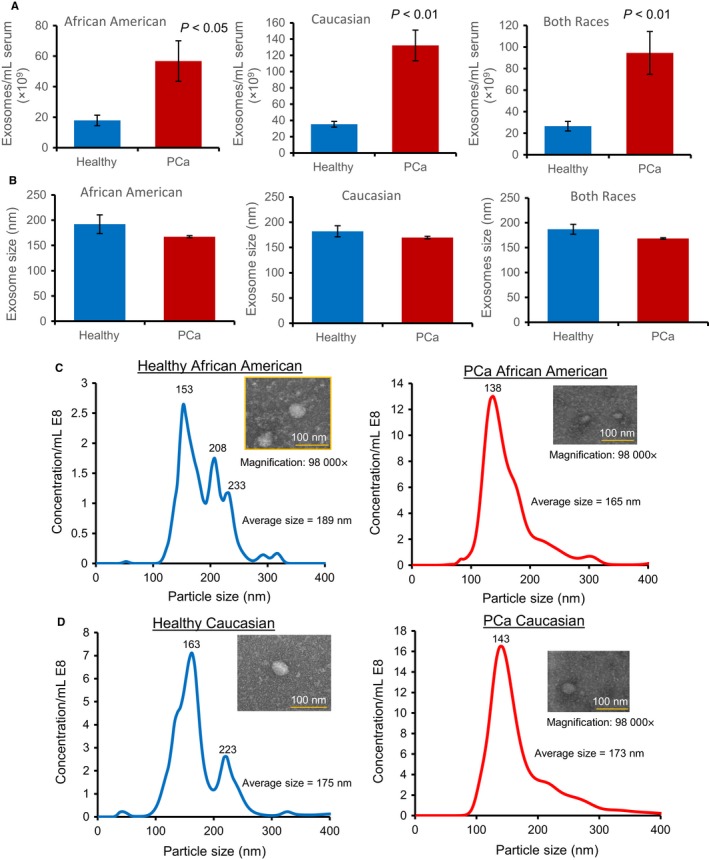
Characterization of exosomes size and concentration by NTA and TEM. Exosomes were isolated by ultracentrifugation method from the serum of African American and Caucasian men either healthy or with PCa. Exosomes size and concentration were characterized by NTA. A, Exosome concentration (number per ml of serum); B, exosome size (nm); C‐D, exosome size distribution and representative TEM images (98 000×) for African American and Caucasian men. Data for A and B represent mean ± SEM of 3‐6 samples

Analysis of clinical serum samples showed that compared to healthy individuals, exosome concentration was 3.5‐fold (*P *˂ 0.01) higher in the sera of PCa patients in both the races (Figure 1A). In the sera of African American PCa patients, the exosome number was 3.2 times (*P *˂ 0.05) more than the healthy African American (Figure 1A). Caucasian PCa patients showed a 3.7‐fold (*P *˂ 0.01) higher exosome number compared with healthy Caucasian (Figure 1A). There was no significant change in the exosome size between healthy and PCa patients (Figure 1B). The size distribution of exosomes in healthy and PCa African Americans is shown in Figure 1C and that of Caucasian in Figure 1D. We also characterized the exosome size by transmission electron microscopy (TEM), and representative images are shown in Figure 1C,D.

### 3.2 Proteomic analysis of exosomes

**Figure 2:**
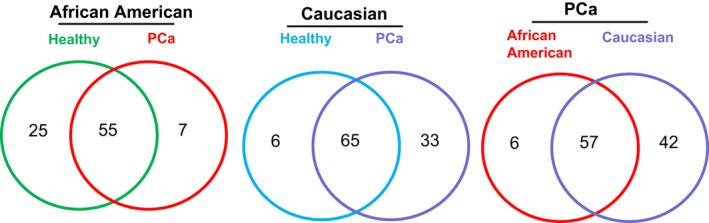
Mass spectrometry analysis of proteins loaded in exosomes. Exosomes isolated from the serum of African American and Caucasian men either healthy or with PCa were analyzed by mass spectrometry. Number of proteins in exosomes from each group is presented by Venn diagram

Proteins loaded in exosomes were characterized by mass spectrometry. The number of proteins shared or uniquely present in different groups is depicted in Figure 2. Serum exosomal proteins showed 55 common proteins in African American men with PCa (PAA) and healthy African American men without PCa (NAA) (Figure 2). Interestingly, seven unique proteins (Isoform 2 of Coiled‐coil and C2 domain‐containing protein 1A, Keratin type I cytoskeletal 10, UPF0728 protein C10orf53, DnaJ homolog subfamily C member 13, Prothrombin, Apolipoprotein(a), and Coiled‐coil domain‐containing protein 172) were present only in PAA and not in NAA (Table 1). The top 10 upregulated proteins in PAA, as compared to NAA, included Keratin type II cytoskeletal 2 epidermal (64‐fold), Serum amyloid P‐component (39‐fold), Keratin type II cytoskeletal 1 (13‐fold), Keratin type I cytoskeletal 9 (12‐fold), Isoform 2 of Filamin A (2.6‐fold), Isoform 3 of Vitamin D‐binding protein (2.4‐fold), Ig kappa chain V‐II region Cum (2.4‐fold), Isoform 2 of Eukaryotic translation initiation factor 2‐alpha kinase 4 (1.8‐fold), Ig alpha‐1 chain C region (1.7‐fold), and Afamin (1.5‐fold) (Table 1). The top 10 downregulated proteins in PAA include Complement component C8 alpha chain (0.07‐fold), Apolipoprotein A‐I (0.04‐fold), Plasminogen (0.08‐fold), Complement component C7 (0.1‐fold), Isoform 2 of Inter‐alpha‐trypsin inhibitor heavy chain H4 (0.2‐fold), Inter‐alpha‐trypsin inhibitor heavy chain H1 (0.2‐fold), Complement C1s subcomponent (0.2‐fold), Ig heavy chain V‐III region BRO (0.2‐fold), Ig gamma‐2 chain C region (0.2‐fold), and Ig gamma‐3 chain C region (0.2‐fold) (Table 1).

**Table 1:** Expression of serum exosomal proteins in African American men with PCa (PAA) compared to healthy African American men (NAA)

	Fold change
Top 10 upregulated proteins in PAA in comparison with NAA	
Keratin, type II cytoskeletal 2 epidermal	64
Serum amyloid P‐component	39
Keratin, type II cytoskeletal 1	13
Keratin, type I cytoskeletal 9	12
Isoform 2 of Filamin A	2.6
Isoform 3 of Vitamin D‐binding protein	2.4
Ig kappa chain V‐II region Cum	2.4
Isoform 2 of Eukaryotic translation initiation factor 2‐alpha kinase 4	1.8
Ig alpha‐1 chain C region	1.7
Afamin	1.5
Top 10 downregulated proteins in PAA in comparison with NAA	
Complement component C8 alpha chain	0.07
Apolipoprotein A‐I	0.04
Plasminogen	0.08
Complement component C7	0.1
Isoform 2 of Inter‐alpha‐trypsin inhibitor heavy chain H4	0.2
Inter‐alpha‐trypsin inhibitor heavy chain H1	0.2
Complement C1s subcomponent	0.2
Ig heavy chain V‐III region BRO	0.2
Ig gamma‐2 chain C region	0.2
Ig gamma‐3 chain C region	0.2
Unique proteins present in PAA as compared to NAA	
Isoform 2 of Coiled‐coil and C2 domain‐containing protein 1A, Keratin, type I cytoskeletal 10, UPF0728 protein C10orf53, DnaJ homolog subfamily C member 13, Prothrombin, Apolipoprotein(a), Coiled‐coil domain‐containing protein 172	

Next, we analyzed the proteins loaded in exosomes from healthy caucasian men without PCa (NCC) and Caucasian men with PCa (PCC) (Table 2). Results showed that there were 65 common proteins in exosomes from NCC and PCC, while 33 unique proteins were loaded in exosomes from PCC (Figure 2A). The top 10 upregulated proteins in exosomes from PCC included Alpha‐1‐acid glycoprotein 1 (13.6‐fold), Alpha‐1B‐glycoprotein (11.6‐fold), von Willebrand factor (5.0‐fold), Ig heavy chain V‐II region NEWM (6.0‐fold), Ig heavy chain V‐III region BRO (5.7‐fold), Afamin (5.4‐fold), Apolipoprotein A‐I (4.9‐fold), Isoform 3 of Vitamin D‐binding protein (4.3‐fold), Complement C1r subcomponent (4.3‐fold), and Angiotensinogen (4.1‐fold) (Table 2). The top 10 downregulated proteins in PAA include SCO‐spondin (0.1‐fold), Complement component C7 (0.1‐fold), Isoform 2 of Ankyrin repeat and SOCS box protein 18 (0.3‐fold), Gelsolin (0.3‐fold), Serum amyloid P‐component (0.4‐fold), Ig heavy chain V‐I region V35 (0.5‐fold), Complement C1q subcomponent subunit C (0.5‐fold), Complement component C8 alpha chain (0.6‐fold), Isoform 2 of Filamin A (0.6‐fold), and Inter‐alpha‐trypsin inhibitor heavy chain H2 (0.7‐fold) (Table 2).

**Table 2:** Expression of serum exosomal proteins in Caucasian men with PCa (PCC) compared to healthy Caucasian men (NCC)

	Fold change
Top 10 upregulated proteins in PCC in comparison with NCC	
Alpha‐1‐acid glycoprotein 1	13.6
Alpha‐1B‐glycoprotein	11.6
von Willebrand factor	5.0
Ig heavy chain V‐II region NEWM	6.0
Ig heavy chain V‐III region BRO	5.7
Afamin	5.4
Apolipoprotein A‐I	4.9
Isoform 3 of Vitamin D‐binding protein	4.3
Complement C1r subcomponent	4.3
Angiotensinogen	4.1
Top 10 downregulated proteins in PCC in comparison to NCC	
SCO‐spondin	0.1
Complement component C7	0.1
Isoform 2 of Ankyrin repeat and SOCS box protein 18	0.3
Gelsolin	0.3
Serum amyloid P‐component	0.4
Ig heavy chain V‐I region V35	0.5
Complement C1q subcomponent subunit C	0.5
Complement component C8 alpha chain	0.6
Isoform 2 of Filamin A	0.6
Inter‐alpha‐trypsin inhibitor heavy chain H2	0.7
Unique proteins present in PCC in comparison with NCC	
Microtubule‐associated protein 10, Prolow‐density lipoprotein receptor‐related protein 1, Ig heavy chain V‐I region HG3, Low‐density lipoprotein receptor‐related protein 1B, Low‐density lipoprotein receptor‐related protein 1B, Ig heavy chain V‐III region KOL, Ig alpha‐2 chain C region, Isoform 8 of Ubiquitin carboxyl‐terminal hydrolase 48, Complement C1q subcomponent subunit B, Ig heavy chain V‐III region GAL, Protein AMBP, Isoform 2 of Plasma protease C1 inhibitor, Isoform 3 of Probable aminopeptidase NPEPL1, Lysine‐specific demethylase 2A, Colipase‐like protein 2, Ig kappa chain V‐III region WOL, Ig kappa chain V‐I region EU, Ig kappa chain V‐II region Cum, Galectin‐3‐binding protein, Complement C4‐B, Vitamin K‐dependent protein S, Complement component C8 beta chain, Isoform 2 of Apolipoprotein L1, Protein Jumonji, Ig lambda chain V‐I region WAH, Heparin cofactor 2, Retinol‐binding protein 4, Isoform 3 of Zinc finger protein 638, Apolipoprotein(a), Signal peptide, CUB and EGF‐like domain‐containing protein 1, Ig heavy chain V‐III region BUT, Isoform 2 of Insulin‐like growth factor‐binding protein complex acid labile subunit, Apolipoprotein E, and Potassium voltage‐gated channel subfamily B member 1	

We further compared proteins loaded in PAA serum exosomes with Gleason score‐matched Caucasian PCa (PCC) serum exosomes (Figure 2). PAA and PCC exosomes shared 57 common proteins, while PAA serum exosomes showed six unique proteins (Isoform 2 of Coiled‐coil and C2 domain‐containing protein 1A, UPF0728 protein C10orf53, Ig kappa chain V‐I region EU, Ig kappa chain V‐II region Cum, DnaJ homolog subfamily C member 13, and Coiled‐coil domain‐containing protein 172). Furthermore, top 10 upregulated proteins in PAA, as compared to PCC, included Isoform 2 of Eukaryotic translation initiation factor 2‐alpha kinase 4 (34‐fold), Keratin type I cytoskeletal 10 (10‐fold), SCO‐spondin (5.3‐fold), Serum amyloid P‐component (fourfold), Apolipoprotein(a) (3.9‐fold), Keratin, type I cytoskeletal 9 (threefold), Ig kappa chain V‐IV region (2.4‐fold), Isoform 2 of Filamin A (2.4‐fold), Keratin, type II cytoskeletal 1 (2.6‐fold), and Keratin, type II cytoskeletal 2 epidermal (2.3‐fold) (Table 3). The top 10 downregulated proteins in PAA, as compared to PCC, included Ig heavy chain V‐III region BRO (0.1‐fold), Alpha‐1‐antitrypsin (0.1‐fold), Plasminogen (0.1‐fold), Ig heavy chain V‐III region TIL (0.1‐fold), Hemopexin (0.1‐fold), Afamin (0.1‐fold), Inter‐alpha‐trypsin inhibitor heavy chain H1 (0.1‐fold), Protein AMBP (0.1‐fold), Alpha‐1B‐glycoprotein (0.03‐fold), and Complement component C8 alpha chain (0.06‐fold) (Table 3).

**Table 3:** Expression of serum exosomal proteins in African American men with PCa (PAA) with respect to Caucasian men with PCa (PCC)

	Fold change
Top 10 upregulated proteins in PAA in comparison with PCC	
Isoform 2 of Eukaryotic translation initiation factor 2‐alpha kinase 4	34
Keratin, type I cytoskeletal 10	10
SCO‐spondin	5.3
Serum amyloid P‐component	4
Apolipoprotein(a)	3.9
Keratin, type I cytoskeletal 9	3
Ig kappa chain V‐IV region	2.4
Isoform 2 of Filamin A	2.4
Keratin, type II cytoskeletal 1	2.6
Keratin, type II cytoskeletal 2 epidermal	2.3
Top 10 downregulated proteins in PAA in comparison with PCC	
Ig heavy chain V‐III region BRO	0.1
Alpha‐1‐antitrypsin	0.1
Plasminogen	0.1
Ig heavy chain V‐III region TIL	0.1
Hemopexin	0.1
Afamin	0.1
Inter‐alpha‐trypsin inhibitor heavy chain H1	0.1
Protein AMBP	0.1
Alpha‐1B‐glycoprotein	0.03
Complement component C8 alpha chain	0.06
Unique proteins present in PAA as compared to PCC	
Coiled‐coil domain‐containing protein 172, DnaJ homolog subfamily C member 13, Ig kappa chain V‐I region EU, UPF0728 protein C10orf53, and Isoform 2 of Coiled‐coil and C2 domain‐containing protein 1A	

### 3.3 Ingenuity pathway analysis of proteomic data

Ingenuity pathway analysis was next performed on 62 proteins present in exosomes isolated from the serum of African American PCa patients. Core analysis in IPA showed that top five canonical pathways include acute‐phase response signaling pathway (*P* = 4.68E−24), Complement System (*P* = 6.55E−14), LXR/RXR Activation (*P* = 5.94E−12), FXR/RXR Activation (*P* = 4.92E−10), and Hematopoiesis from Pluripotent Stem Cells (*P* = 6.20E−07) (Table 4). IPA also identified the top upstream regulators and top diseases and biofunctions (described in Table 4). Similar IPA analysis of 98 proteins present in exosomes from Caucasian PCa patients is described in Table S1.

**Table 4:** IPA analysis of exosome proteins present in African American men with PCa

	*P*‐value
Top canonical pathways	
Acute‐phase response signaling	4.68E−24
Complement System	6.55E−14
LXR/RXR Activation	5.94E−12
FXR/RXR Activation	4.92E−10
Hematopoiesis from Pluripotent Stem Cells	6.20E−07
Top upstream regulators	
HNF1A	3.86E−10
IL6	2.82E−09
CEBPB	8.50E−08
Nitrofurantoin	1.22E−07
Lipopolysaccharide	2.75E−07
Top diseases and biofunctions	
Diseases and disorders	
Developmental disorder	1.46E−03‐6.84E−14
Hereditary disorder	1.46E−03‐6.84E−14
Immunological disease	1.45E−03‐6.84E−14
Organismal injury and abnormalities	1.46E−03‐6.84E−14
Neurological disease	1.45E−03‐6.36E−12
Molecular and cellular functions	
Cell‐to‐cell signaling and interaction	1.46E−03‐1.68E−09
Molecular transport	1.46E−03‐1.97E−08
Cellular assembly and organization	1.45E−03‐9.65E−08
Cell death and survival	1.45E−03‐1.54E−07
Cellular compromise	1.45E−03‐2.88E−07
Physiological system development and function	
Humoral immune response	1.45E−03‐7.49E−11
Hematological system development and function	1.45E−03‐1.68E−09
Immune cell trafficking	1.45E−03‐2.01E−08
Tissue morphology	1.45E−03‐1.41E−07
Tissue development	1.46E−03‐1.67E−07

### 3.4 Exosomes from African American PCa E006AA‐hT cells promote pro‐inflammatory phenotype in macrophages and sensory neurons

Ingenuity pathway analysis analyses of exosomal proteins identified acute‐phase response signaling as the top pathway in PAA. To further confirm that, next, we assessed the effect of exosomes secreted by African American PCa E006AA‐hT cells under normoxic and hypoxic conditions on inflammatory phenotype in cell culture models.

**Figure 3:**
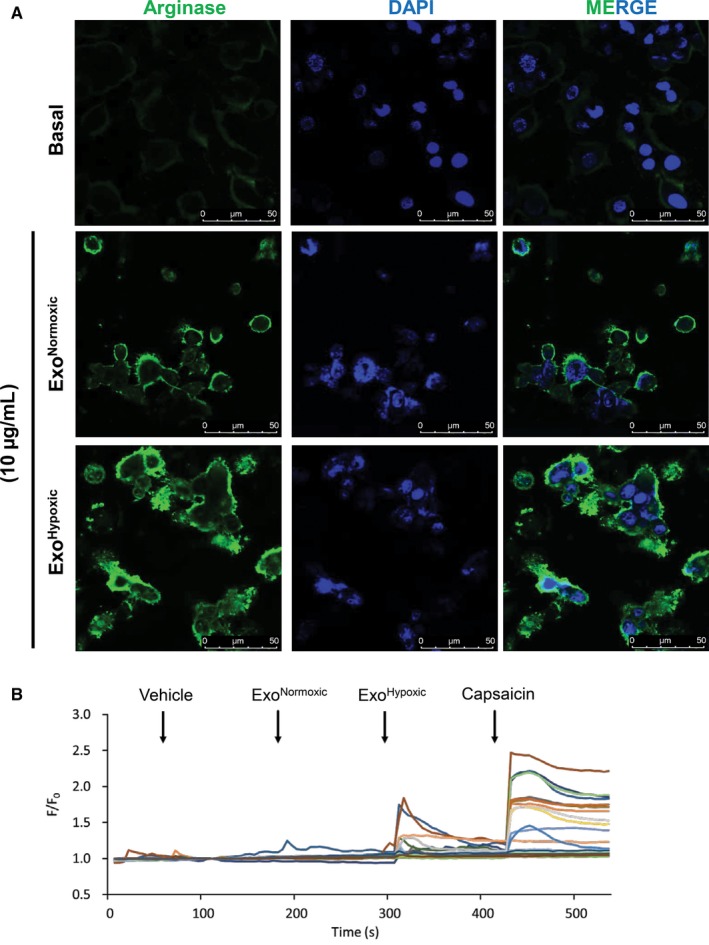
Exosomes from African American PCa E006AA‐hT cells promote proinflammatory phenotype in macrophages and sensory neurons. A, THP1 cells were cultured in chambered coverslip along with PMA (100 ng/mL) for 12 h followed by treatment with Exo^Normoxic^ or Exo^Hypoxic^ (10 µg) and processed for arginase‐1 expression (green) by confocal microscopy. DAPI (blue) was used to stain nuclei. Representative images are shown at 60×. B, A change in intracellular calcium concentration of cultured DRG neurons to exosomes was measured by Ca^2+^ imaging. Representative traces of DRG neurons stimulated with Exo^Normoxic^ and Exo^Hypoxic^ (10 µg/mL) are presented. Rapid and transient cytosolic Ca^2+^ elevations were recorded, which was evoked by successive application (vertical arrow) of vehicle, Exo^Normoxic^ and Exo^Hypoxic^ (10 μg/mL each) and 1 μmol/L capsaicin. N = 30 neurons

M2 phenotype of macrophage is associated with inflammation. We treated macrophages‐derived from THP1 monocytes with exosome secreted by E006AA‐hT cells under normoxic (Exo^Normoxic^) and hypoxic (Exo^Hypoxic^) conditions. As shown in Figure 3A, both Exo^Normoxic^ and Exo^Hypoxic^ treatment strongly induced the arginase expression in macrophages, suggesting an induction of M2 type.

Next, to determine the effect of Exo^Normoxic^ and Exo^Hypoxic^ on inflammation, we performed calcium imaging using primary cultured mouse dorsal root ganglion (DRG) neurons. We successively applied bath solution as vehicle, 10 μg/mL of Exo^Normoxic^, 10 μg/mL of Exo^Hypoxic^, and 1 μmol/L capsaicin. Interestingly, Exo^Hypoxic^ but not Exo^Normoxic^ induced cytosolic Ca^2+^ increases in DRG neurons, majority of which were responding to capsaicin as well. Capsaicin is known as one of most potent algogens to evoke strong pain through transient receptor potential vanilloid 1 (TRPV1), which shows relatively broad expression in human and mouse DRG. Our results suggest that the Exo^Hypoxic^ directly activates primary sensory neurons and might induce pain, some extent of which might be mediated by TRPV1.

### 3.5 Filamin A expression in PCa datasets

**Figure 4:**
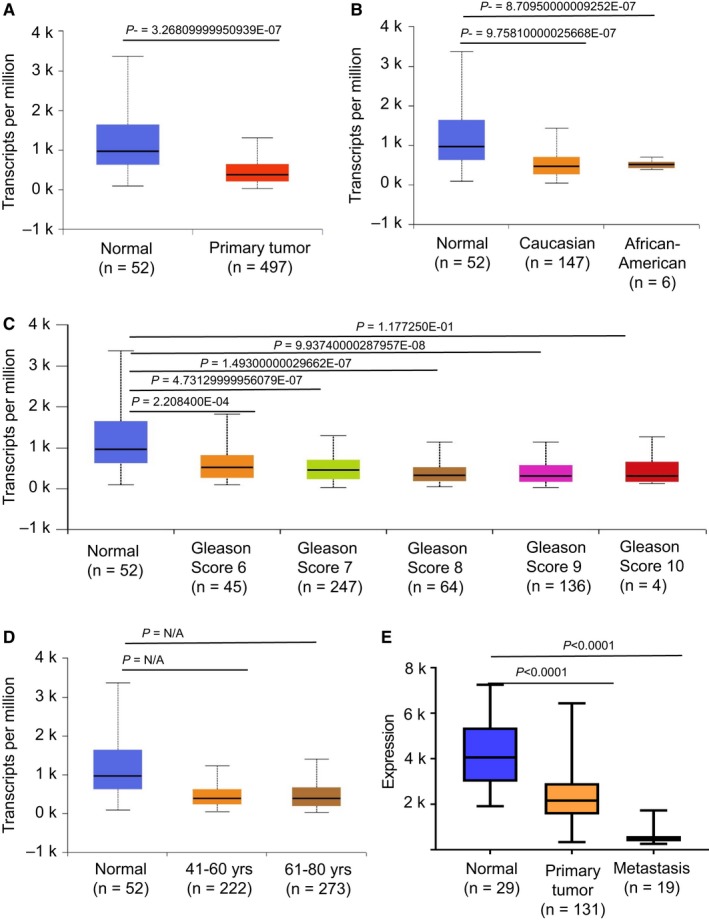
Filamin A expression in PCa determined through TCGA database and Taylor's cohort. Box‐whisker plots showing the expression of Filamin A in TCGA based on (A) normal versus primary tumor, (B) patient's race, (C) patient's Gleason score, and (D) patient's age. (E) Box‐whisker plots showing the expression of Filamin A in Taylor's cohort based on major cancer stages

We identified Filamin A as one of the differentially loaded proteins in PAA exosomes, so we further assessed its expression in available PCa datasets. We analyzed TCGA gene expression data for Filamin A using UALCAN, an interactive web‐portal uses TCGA level 3 RNA‐seq and clinical data from 31 cancer types.^32^ Data indicate that Filamin A transcript is downregulated in primary tumor compared with normal prostate tissue (Figure 4A). There is no significant difference observed in the Filamin A expression between races (Figure 4B), among different Gleason score disease (Figure 4C), or age (Figure 4D). We also observed a decrease in the Filamin A expression in primary tumor and metastatic samples in Taylor's cohort (Figure 4E).

### 3.6 Filamin A expression in PCa tissues

**Figure 5:**
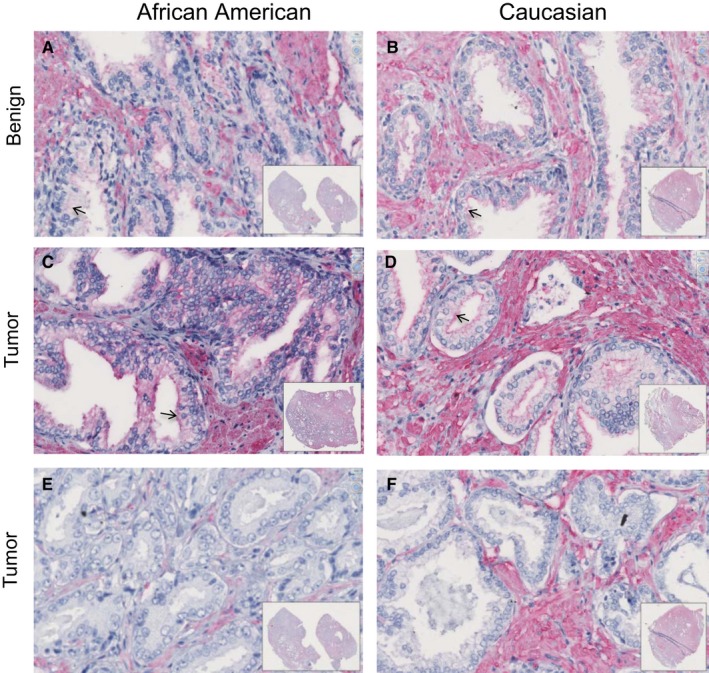
Filamin A expression in PCa tissues. PCa tissue sections from African American and Caucasian men were stained for Filamin A expression by IHC as described in methods, and representative photomicrographs are shown. A,B, Filamin A staining in benign prostate glands; C‐F, images for Filamin A staining in tumor area

Next, we assessed Filamin A expression in prostatectomy tissue sections. Filamin A expression was observed in 25% (2/8) of tumors in both African American (n = 8) and Caucasian patients (n = 8) with prostatic adenocarcinoma (Table S2). The expression was variable and typically seen in the cytoplasm. In the cancerous glands, Filamin A expression was either patchy or focal with weak to moderate intensity (Figure 5). The positivity was more on luminal aspect (arrow) in one of the patients of each group. The overall Filamin A expression was slightly more in prostatic adenocarcinoma of African American patients than that of Caucasian patients (Table S2). Majority of the prostate cancers (75%), however, showed no Filamin A expression. The benign prostate glands also showed weak to moderate cytoplasmic immunoreactivity, and the distribution was patchy similar to cancer glands (Figure 5). About 62.5% of African American (n = 8) and all cases of Caucasian patients showed mild to moderate staining in benign glands at least in focal or patchy areas. Cytoplasmic staining with luminal accentuation (arrow) was observed in approximately 37.5% cases of African American (n = 8) and 37.5% of Caucasian patients (n = 8) (Table S2). No nuclear positivity was seen in the malignant or benign glands. Filamin A was strongly positive in stromal components such as smooth muscle, fibroblastic cells, and smooth muscle cells of blood vessels.

## 4 DISCUSSION

Discovery of novel biomarker from exosomes is an exciting approach, since the cargo of exosomes is well protected by lipid bilayer and they often have a distinctive cargo (eg, proteins, RNAs, lipids, metabolites) dependent upon the cell of their origin.^20^, ^25^, ^30^, ^33^, ^34^, ^35^ Furthermore, compared to the complex environment of plasma, exosomes offer a much cleaner clinical sample devoid of abundant plasma proteins, facilitating analyses.^36^ In the present study, we characterized the exosomal proteins from healthy and PCa patients both from African American and Caucasian races. We observed distinct signature proteins in African American PCa exosomes compared with Caucasians. Some of these proteins have already been studied for their biomarker application in other cancers. For example, Filamin A has earlier been studied as a biomarker in breast cancer,^37^ Vitamin D‐binding protein in pancreatic cancer,^38^ and Afamin in ovarian cancer.^39^ Interestingly, here, we report that these proteins are present in exosomes from African Americans with PCa.

Although in the literature, there is already one study regarding exosomal proteomic analysis in ethnically diverse PCa patients,^40^ our study is different due to the following reasons: We have (a) characterized the exosomes from serum, (b) isolated exosomes by classical ultracentrifugation method, (c) quantified the exosomes by NTA, and (d) provided relative quantification of exosomal proteins (in terms of fold change). More importantly, in the present study, we have matched PCa with healthy individual for each race. The earlier study^40^ characterized the exosomes from plasma, isolated the exosomes by ExoQuick method, quantified the exosomes by acetylcholinesterase activity assay, and reported the presence of exosomal proteins without fold change. In addition, they bundled healthy individual groups from all the races.

In the present study, proteomic analysis and subsequent IPA analysis of proteins loaded in exosomes revealed a strong association with the acute‐phase response signaling pathway. The acute‐phase response is a rapid inflammatory response that generally provides protection against microorganisms using nonspecific defense mechanisms.^41^ In agreement with our results, Davalieva et al^42^ reported acute‐phase response proteins as candidate diagnostic biomarkers for PCa by proteomic analysis of urine samples. Further, our results suggest that the higher systemic inflammation in African Americans could be due to exosomes secreted by the prostate tumor cells, as exosomes secreted by African American PCa E006AA‐hT cells promoted the M2 phenotype in macrophages. Macrophages are component of innate immunity, and growing tumors might educate the macrophages to its advantage via promoting the proinflammatory phenotype in macrophages. Furthermore, exosomes secreted by African American PCa cells also promoted the neuron excitation similar to that induced by capsaicin, an agonist for TRPV1‐receptor, whose activation causes pain response in mice.^43^ Interestingly, our results suggest a possible role of exosomes from hypoxic African American PCa E006AA‐hT cells in producing pain by some unknown mechanisms, which is yet to be determined. In addition, IPA analysis showing significant upregulated signals associated with lipopolysaccharides (LPS) raises the possibility that LPS from gut microbiome may contribute in systemic inflammation in PCa patients. Overall, these results support the further development of exosomes to noninvasively assess inflammatory phenotype in PCa patients and decide the usefulness of anti‐inflammatory agents.

Filamins are a family of cytoskeletal proteins that organize filamentous actin into networks and stress fibers.^44^ Filamin A dimerization forms a V‐shaped flexible structure which can induce high‐angle orthogonal branching and efficiently gather actin filaments into a three‐dimensional gel in vitro by cross‐linking actin filaments at the leading edge of migrating cells. Hence, Filamins are essential for mammalian cell locomotion, anchoring of transmembrane proteins including integrins, and also act as interfaces for protein‐protein interaction.^28^

Several studies indicate the correlation of Filamin A with cancer stages and patient prognosis.^29^ Bedolla et al^45^ have shown by immunohistochemical examination in paraffin‐embedded human prostate tissues, that benign prostate, PIN, and localized PCa have predominantly nuclear Filamin A expression, whereas in metastatic PCa, Filamin A was found to be primarily in the cytoplasm. Another study detected both Filamin A and Filamin B in the plasma from men with suspected PCa.^46^ Recently, the role of Filamin A has been mentioned as a new tumor suppressor gene for colorectal adenocarcinoma.^47^ In the present study, by proteomic analysis of exosomes, we observed a higher level of Filamin A in African American PCa patients compared with Caucasian patients. However, we observed a decrease in Filamin A expression in prostatic adenocarcinoma as compared to benign or normal glands in both African American and Caucasian patients. The similar decreased expression of Filamin A in PCa compared to normal tissue was observed in TCGA and Taylor's PCa dataset. These observations indicate that the Filamin A decrease in PCa cells could be through its increased secretion via exosomes in African Americans. While several mechanisms exist for regulating the gene expression such as mutations, epigenetic silencing, or posttranslational degradation, cellular expression regulation through exosomes secretion could be a novel mechanism and should be investigated further. In agreement with our hypothesis, Gabriel et al (2013) have detected the tumor suppressor PTEN in the PCa patients’ blood exosomes, whereas normal subjects have no PTEN expression in their blood exosomes.^48^

## 5 CONCLUSION

Serum exosome analyses could offer useful information about primary PCa and could be important for diagnosis and prognosis of the disease.
