# Supplementary material for: Exosome proteomic analyses identify inflammatory phenotype and novel biomarkers in African American prostate cancer patients
Source: Cancer Med. 2019 Jan 8;8(3):1110–23. doi: 10.1002/cam4.1885 (PMC6434210; doi:10.1002/cam4.1885)
Supplement: Supplementary file 1 [file CAM4-8-1110-s001.docx]

**Table S1:** IPA analysis of exosome proteins present in Caucasian PCa patients

| **Top Canonical Pathways** | **p-value** |
| --- | --- |
| Acute Phase Response Signaling | 1.28E-31 |
| Complement System | 1.15E-23 |
| LXR/RXR Activation | 1.28E-20 |
| FXR/RXR Activation | 1.64E-18 |
| Coagulation System | 1.86E-10 |
| **Top Upstream Regulators** | **p-value** |
| HNF1A | 1.41E-11 |
| Inosine | 2.20E-11 |
| Nitrofurantoin | 1.24E-10 |
| Methotrexate | 1.59E-10 |
| Lipopolysaccharide | 3.05E-10 |
| **Top Diseases and Bio Functions** |  |
| **Diseases and Disorders** | **p-value** |
| Developmental Disorder | 1.30E-04 - 3.64E-23 |
| Hereditary Disorder | 1.30E-04 - 3.64E-23 |
| Immunological Disease | 1.77E-04 - 3.64E-23 |
| Organismal Injury and Abnormalities | 2.75E-04 - 3.64E-23 |
| Neurological Disease | 2.73E-04 - 1.98E-16 |
| **Molecular and Cellular Functions** | **p-value** |
| Cell-To-Cell Signaling and Interaction | 2.60E-04 - 1.82E-13 |
| Cellular Compromise | 1.83E-05 - 3.71E-10 |
| Cellular Function and Maintenance | 2.06E-04 - 3.71E-10 |
| Cellular Movement | 2.65E-04 - 1.33E-09 |
| Molecular Transport | 2.55E-04 - 2.83E-09 |
| **Physiological System Development and Function** | **p-value** |
| Humoral Immune Response | 2.33E-04 - 1.89E-15 |
| Hematological System Development and Function | 2.65E-04 - 1.82E-13 |
| Immune Cell Trafficking | 2.65E-04 - 1.82E-13 |
| Cardiovascular System Development and Function | 2.75E-04 - 2.20E-11 |
| Organismal Development | 2.75E-04 - 2.20E-11 |

**Table S2:** Detail of Filamin A expression in prostatectomy sections from African American (AA) and Caucasian (CA) PCa patients.

| **Patient** | **In tumor** | **In benign glands** | **In stroma** | **Lymphocytes** | **Blood vessels** | **Nerve** | **Ganglion cells** |
| --- | --- | --- | --- | --- | --- | --- | --- |
| AA | 0 | 0 | 3+ | 0 | 3+ | Weakly positive | Not seen |
| AA | 0 | 0 | 3+ | 0 | 3+ | 0 | 0 |
| AA | 0 | 0 | 3+ | 0 | 3+ | 0 | Not seen |
| AA | 0 | Patchy 2+ (luminal +) | 3+ | 0 | 3+ | Weakly positive | Not seen |
| AA | 0 | 1+ (luminal +) | 3+ | 0 | 3+ | 0 | Not seen |
| AA | Focal 1+ | 1+, focal 2+ | 3+ | 0 | 3+ | 0 | Not seen |
| AA | Patchy 2-3+ (luminal +) | 1-2+, focal 3+ (luminal +) | 3+ | 0 | 3+ | Nerve not seen | Not seen |
| AA | 0 | Patchy 1-2+ | 3+ | 0 | 3+ | 0 | 0 |
| CA | 0 | 1+ | 3+ | 0 | 3+ | Nerve not seen | Not seen |
| CA | 0 | 1+ | 3+ | 0 | 3+ | 0 | 0 |
| CA | 0 | 1+, focal 2+ | 3+ | 0 | 3+ | Nerve not seen | Not seen |
| CA | 0 | Patchy 1+, (luminal +) | 3+ | 0 | 3+ | 0 | Not seen |
| CA | 0 | 1+ | 3+ | 0 | 3+ | 0 | Not seen |
| CA | 0 | Patchy 1+, focal 2+ | 3+ | 0 | 3+ | Nerve not seen | Not seen |
| CA | 1+, (luminal +) | 1+ (luminal more +) | 3+ | 0 | 3+ | 0 | Not seen |
| CA | Focal 1+ | Patchy 1+, (luminal +) | 3+ | 0 | 3+ | 0 | Not seen |
